# Genetic Structure of *Daphnia galeata* Populations in Eastern China

**DOI:** 10.1371/journal.pone.0120168

**Published:** 2015-03-13

**Authors:** Wenzhi Wei, Sabine Gießler, Justyna Wolinska, Xiaolin Ma, Zhong Yang, Wei Hu, Mingbo Yin

**Affiliations:** 1 Yangzhou University, College of Animal Science and Technology, Yangzhou, China; 2 Ludwig-Maximilians-Universität, Department Biologie II, Evolutionsökologie, Planegg-Martinsried, Germany; 3 Leibniz-Institute of Freshwater Ecology and Inland Fisheries, Department of Ecosystem Research, Berlin, Germany; 4 Fudan University, School of Life Science, MOE Key Laboratory for Biodiversity Science and Ecological Engineering, Shanghai, China; East China Normal University, CHINA

## Abstract

This study presents the first examination of the genetic structure of *Daphnia longispina* complex populations in Eastern China. Only one species, *D*. *galeata*, was present across the eight investigated lakes; as identified by taxon assignment using allelic variation at 15 microsatellite loci. Three genetically differentiated *D*. *galeata* subgroups emerged independent of the type of statistical analysis applied. Thus, Bayesian clustering, discriminant analysis based on results from factorial correspondence analysis, and UPGMA clustering consistently showed that populations from two neighbouring lakes were genetically separated from a mixture of genotypes found in other lakes, which formed another two subgroups. Clonal diversity was high in all *D*. *galeata* populations, and most samples showed no deviation from Hardy-Weinberg equilibrium, indicating that clonal selection had little effect on the genetic diversity. Overall, populations did not cluster by geographical origin. Further studies will show if the observed pattern can be explained by natural colonization processes or by recent anthropogenic impact on predominantly artificial lakes.

## Introduction

Cyclically parthenogenetic *Daphnia* (Crustacea: Anomopoda) are present in a wide range of different water bodies [1], and are a key component of freshwater ecosystems (e.g. [2,3]). *Daphnia galeata* G. O. Sars, 1863, a species belonging to the *D*. *longispina* complex (taxonomy revised in [4]), has a wide Holarctic distribution [5]. This species inhabits freshwater lakes and ponds in Europe, North America and Asia (e.g. [6,7–9]). While *D*. *galeata* is typically detected in warm and eutrophic lakes (e.g. [7,10]), it has also been found in alpine habitats with lower trophic level (e.g. [11]). This species often coexists with some other members of the *D*. *longispina* complex (e.g. [6,8,10]). In such cases, *D*. *galeata* is usually involved in local hybridizations with *D*. *cucullata*, *D*. *dentifera* or *D*. *longispina* (e.g. [10,12,13,14]), with interspecific hybrids sometimes reaching high abundances (e.g. [7,10]).

*Daphnia* are cyclical parthenogens, meaning that most of the time females clone themselves, producing parthenogenetic daughters. During unfavourable periods, however, individuals switch to the production of males and sexual haploid eggs which must be fertilized [15]. These fertilized eggs are then released and sink to the bottom of a lake where they form an egg bank. The sexual phase can be triggered by a lack of food, overcrowding, or low temperature (e.g. [16]). Such eggs, known as diapause eggs, allow the persistence of a population across the seasons as well as the ability for populations to disperse into new habitats [17], even on intercontinental scales (e.g. [18]). Wind and birds are believed to be the main vectors for the passive dispersal of diapause eggs (e.g. [19,20]). The dispersal capabilities of *D*. *galeata* are high (e.g. [17,21]), resulting in rapid colonisation of new habitats (e.g. [21,22]). In contrast to its high dispersal capacity, however, *D*. *galeata* has been found to exhibit strong population genetic differentiation, even over small geographical scales (reviewed in [23]).

The *D*. *longispina* complex has been recorded across China in the 1970s, and several members from this species complex have been described to coexist, based on morphological assignment [24]. Specifically, *D*. *galeata* was reported to have a broad geographical distribution, covering lowland China, and to coexist with *D*. *longispina* in the eastern lakes (e.g. in Jiangsu Province) [24]. Since the species from the *D*. *longispina* complex are characterized by high morphological plasticity (e.g. [25,26]) and because of the possibility of hybridization and introgression (e.g. [8,10,27,28]), morphology-based taxonomy is insufficient for distinguishing genetic units. Genetically based studies are therefore required to reliably explore the distribution of the *D*. *longispina* complex in China. Recently, a large set of microsatellite markers has been developed for the *D*. *longispina* complex [29], and has been applied to the study of the genetic composition of egg banks [30,31], the detection of hybridization (e.g. [10,32]), or the exploration of the population structure of species involved [33,34]. However, to date, no study has investigated the distribution and genetic population structure of the *D*. *longispina* complex from China.

In the present work, we sampled and genotyped (at 15 microsatellite loci) populations from the *D*. *longispina* complex, originating from eight lakes in Eastern China. First, we verified how many species of the *D*. *longispina* complex were present in our samples. We expected to detect several members of *D*. *longispina* complex coexisting in the investigated area, as it was observed in 1970s [24]. We then investigated the genetic structure of the sampled *Daphnia* populations. Our hypothesis here was to test if the *Daphnia* assemblages can be associated to single lakes, and strong population genetic differentiation would be present, as it is typically observed in populations from lakes with a long natural history (reviewed in [23]).

## Materials and Methods

### Ethics statement

Collection of zooplankton (*Daphnia*) in this study did not require specific permissions, and our study did not involve the use or collection of endangered or protected species.

### *Daphnia* collections

Zooplankton samples were collected from twenty lakes (natural lakes or man-made reservoirs) in and around Yangzhou City (Jiangsu province, China). Each lake was sampled once; in late spring or early autumn of 2012 or 2013. Zooplankton samples were collected with a 125-μm plankton net hauled through the whole water column at several different sites per lake. Samples we pooled per lake and preserved in 95% ethanol. Using a stereomicroscope, individuals from the *Daphnia longispina* complex [6] were detected in eight out of twenty sampled water bodies. For each of those eight lakes, about 40 adult *Daphnia* females were then randomly selected for genotyping (313 individuals in total). A list of the eight lakes is provided in Table 1, and their geographical locations are shown on S1 Fig. Today, the eight lakes are not connected to each other. However, in former times a small stream (most of time dry) between BYH and HZH enabled exchange during periods of flooding; since 2000, a road on a dam isolates the two neighbouring lakes. The minimal distance between the lakes is about 4.2 km (between HWB and SHR), and the maximum distance between the lakes is about 291 km (between LMH and ZSR). The remaining twelve lakes (i.e. those where no *D*. *longispina* complex were found during sampling) are listed in S1 Table.

**Table 1:** Genetic diversity of *Daphnia galeata* populations from Eastern China, based on 15 microsatellite loci.

Lake (abbreviation)	Latitude, longitude	Surface area (km^2^)	Origin	Sampling period	N	N*	MLG	Na	Ho	He	Clonal richness	Clonal diversity	HWE	*F*_*IS*_
Baoying Hu (BYH)	33°10′, 119°14′	192.1	Natural	Spring^2012^	39	34	34	80	0.48	0.43	1.00	0.97	0.23	-0.09
Hewangba Reservoir (HWB)	32°32′, 118°50′	35.1	Artificial	Spring^2013^	40	35	17	42	0.33	0.31	0.47	0.88	0.79	-0.08
Hung-tse Lake (HZH)	33°23′, 118°31′	1576.9	Natural	Spring^2012^	44	40	29	58	0.52	0.40	0.72	0.95	***	-0.29
Jinniushan Reservoir (JNS)	32°28′, 118°57′	124.1	Artificial	Spring^2013^	42	37	18	41	0.35	0.27	0.47	0.86	***	-0.35
Luoma Hu (LMH)	34°07′, 118°11′	260.0	Natural	Autumn^2012^	43	33	33	62	0.37	0.40	1.00	0.97	0.95	0.04
Shanhu Reservoir (SHR)	32°26′, 118°47′	30.8	Artificial	Spring^2013^	22	17	17	40	0.29	0.29	1.00	0.95	0.33	-0.001
Zaolin Reservoir (ZLR)	32°20′, 119°04′	6.0	Artificial	Spring^2013^	42	34	31	45	0.32	0.32	0.91	0.96	0.54	-0.05
Zhongshan Reservoir (ZSR)	31°63′, 119°07′	32.3	Artificial	Autumn^2012^	41	34	22	38	0.27	0.28	0.64	0.92	0.64	-0.01

N, Total number of individuals; N*, Number of individuals excluding those lacking a complete multilocus genotype, i.e. all 15 loci; MLG, number of unique multi-locus genotypes; *N*_*a*_, number of alleles, *H*_*o*_, observed heterozygosity; *H*_*e*_, expected heterozygosity; HWE, Hardy-Weinberg-Equilibrium; *F*_*IS*_, inbreeding coefficient; *** P < 0.001.

### DNA extraction and genotyping

The DNA extraction for each *Daphnia* individual followed the protocol reported in Yin *et al*. [10]. DNA was then briefly centrifuged and stored at 4°C. Fifteen microsatellites [29] were amplified in two multiplex polymerase chain reactions [10]. The PCR products were then analysed on an ABI PRISM 3730 capillary sequencer, using a LIZ 500 labelled size standard. Genotypes were scored using GeneMapper version 3.7 (Applied Biosystems), and the alleles at each locus were defined by their fragment length (in base pairs). We used a European reference clone (G100) in each run to check the consistency of alleles before data sets from different plates and European reference clones were merged. All microsatellite markers passed the test for the absence of null alleles, using MICRO-CHECKER [35].

### Species assignment

For genetic analyses of multispecies complexes, statistical methods are needed that allow the classification of individuals to more than two species and their interspecific hybrids. By employing multivariate statistics such as Factorial Correspondence Analysis (FCA), complex relationships among multilocus genotypes (MLGs) can be disentangled by reducing dimensions and calculating factorial axis scores. From the plot of the first two dimensions, the number of emerging groups can be determined and used in subsequent discriminant analysis. By Canonical Discriminant analysis on obtained scores, discriminant functions can be derived, which best predict the group membership of individuals, by the input variables (FCA-scores). Accordingly, in a first step, FCA was applied in GENETIX 4.05 [36] on MLGs based on 15 microsatellite loci; for Chinese *Daphnia* samples combined with data from 49 well-defined reference genotypes, covering five taxa: three parental species (*D*. *cucullata*, *D*. *galeata* and *D*. *longispina*) and two hybrid types (F1 *D*. *cucullata × D*. *galeata* and F1 *D*. *galeata × D*. *longispina*). These genotypes, originating from Europe (except one *D*. *galeata* from North America), had been assigned to species using morphology, allozymes and Mt-DNA (12S and/or Cytb). For a list of reference genotypes, see Yin *et al*. [10]. If data at up to four loci were missing, these individuals were still included. Only unique MLGs were used as input for FCA. Canonical discriminant (CD) analysis on obtained FCA-scores (DFCA) was run in SPSS 20.0 (stepwise method, Mahalanobis distance). Mean discriminant scores for grouping variables (group centroids, here representing taxonomic units) were calculated for each function to examine the pairwise distances of centroids which are correlated with misclassification (the closer the centroids the more likely the misclassification to respective taxonomic units). With focus on the assignment of Chinese MLGs to a certain taxonomic unit, reference genotypes representing two species (*D*. *galeata* and *D*. *longispina*) were selected as prior groups to test the probability of each MLG from the Chinese samples to belong to a particular species. This was done by the Bayesian assignment method of Rannla and Mountain [37], as implemented in GENECLASS 2 [38].

### Population structure

To explore the genetic relationship of MLGs from the eight different Chinese lakes, a similar FCA was run as described above, but without inclusion of the reference clones. Again, in a second step, FCA scores were used in discriminant analysis (here; with the lake as grouping factor: eight groups) to investigate the differentiation among *Daphnia* assemblages from various lakes. To corroborate the results, a Bayesian clustering algorithm was applied in STRUCTURE V2.3.4 [39] which assigns individuals to subgroups (clusters) that have distinctive allele frequencies, assuming the existence of *K* populations or groups. To test also for potential substructure within lakes the twofold number of groups than the number of lakes (i.e. 8) was chosen in the analysis as the maximum possible number of clusters. For each tested value of *K* (i.e. 1 to 16), 15 independent runs were performed and, for each run, 100,000 iterations were carried out after a burn-in period of 10,000 iterations. The most likely *K* was determined by the distribution of *ΔK*, following the methods of Evanno *et al*. [40]. Additionally, pairwise Nei’s distances [41] calculated in GENALEX 6 [42] served to cluster populations by genetic similarities using the Unweighted Pair-Group Method with Arithmetic Means (UPGMA) in MEGA 4 [43]. Finally, to estimate exchange among conspecific *Daphnia* populations, the differentiation coefficient between each pair of populations (*F*_*ST*_) was calculated in Arlequin 3.0 (10^4^ permutations, [44]).

To determine the likelihood that an MLG encountered more than once was the result of sexual recombination, rather than clonal propagation, the *P*_sex_ index [45] was calculated per lake in GENCLONE 2.0 [46]. In this analysis (as well as in the calculations of relative clonal richness and clonal diversity, see below), only individuals characterized at all 15 microsatellite loci were included (i.e. 264 of 313 genotyped *Daphnia*, see Table 1). The level of genetic diversity was evaluated per population by calculating the number of alleles (*N*_*a*_), observed heterozygosity (*H*_*o*_) and expected heterozygosity (*H*_*e*_), using GENEALEX 6 [42]. The deviation from Hardy-Weinberg equilibrium (HWE) was examined in GenePop 3.4 [47]. The inbreeding coefficient (*F*_*IS*_), which measures the extend of non-random mating within populations, was calculated by permuting the alleles among individuals within population in Arlequin [44]. *F*_IS_ ranges from-1 to 1, where negative values indicate an excess of heterozygotes, positive values indicate a deficiency of heterozygotes, and value of zero indicates no deviation from HWE. Moreover, relative clonal richness (R) was calculated per sample as R = (G-1) / (N-1) [48], where G is the number of genotypes and N indicates sample size. The clonal diversity, as a complement of the maximum likelihood estimator of Simpson’s index (1-D) [49], was calculated in SPADE [50].

## Results

### Species assignment

**Fig 1:**
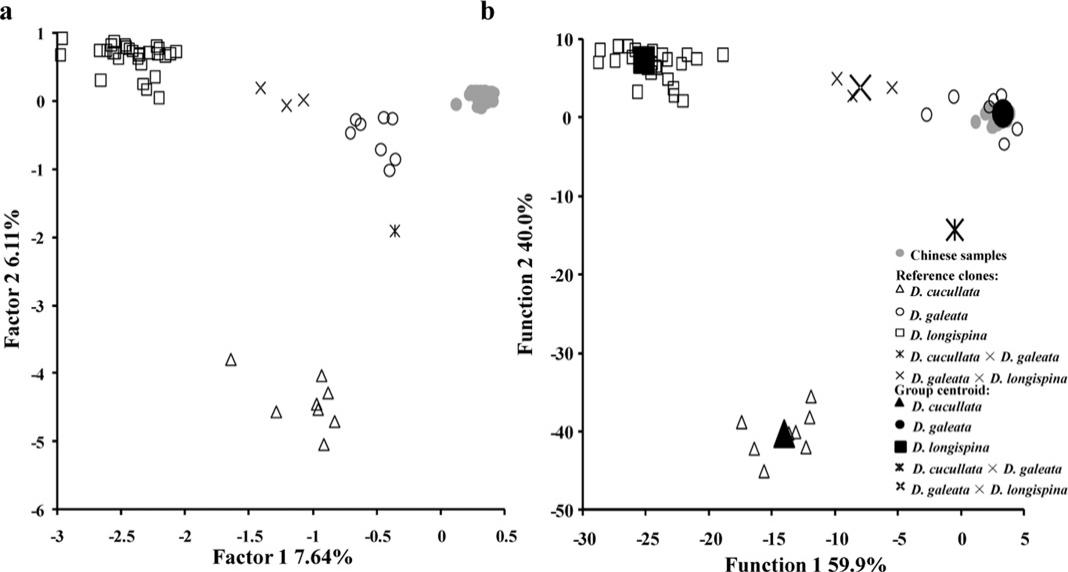
Taxon identity of individuals from the *D*. *longispina* complex sampled from eight lakes in Eastern China. (a) Similarity of Chinese samples to 49 reference clones representing three species and two hybrid taxa (indicated by crosses; for a list of all reference clones see Yin *et al*. 2010). Factorial correspondence analysis scores from the first two axes are shown. FCA based on allelic variation at up to 15 microsatellite loci was used to extract factorial axes. (b) Individuals were reclassified by discriminant functions to taxa using FCA scores from four axes to discriminate among groups. Shown are values from the first two discriminant functions per individual and five group centroids representing the five taxa.

Among 313 individuals from eight lakes in and around Yangzhou city (allowing missing data at up to four loci), 250 had a unique MLG genotype. Those unique MLGs were used in species assignment tests by DFCA. Altogether, ten factorial axes were extracted by FCA (cut-off: eigenvalue > 0.1) in a joint analysis with 49 well-defined reference genotypes [10]. Although the eigenvalues of the first two factorial axes were relatively low (0.73 and 0.58) and only 13.75% of the variance was explained, the plot of respective factorial axis scores resolved at least three species clusters (Fig. 1a). The Chinese samples clustered nearby the *D*. *galeata* reference MLGs but away from two hybrid clusters. When individuals were classified to five groups corresponding to the five reference taxa (*D*. *cucullata*, *D*. *galeata*, *D*. *longispina*, F1 *D*. *cucullata × D*. *galeata* and F1 *D*. *galeata × D*. *longispina*) discriminant analysis on all ten FCA scores confirmed the *D*. *galeata* species identity of the Chinese samples (Fig. 1b). Wilks´ lambdas from the first three discriminant functions were significant (*P* < 0.001). Only the first two functions had eigenvalues > 1 (74.72 and 49.87) and explained 99.8% of the variance in the data. Group centroids of taxonomic units were clearly separated and all Chinese samples unambiguously clustered with eight reference *D*. *galeata* genotypes from Europe and one from North America. Also, when using GENECLASS to assign individuals to one of two species (*D*. *galeata* and *D*. *longispina*) all Chinese *Daphnia* were clearly assigned to *D*. *galeata* (p > 99.7).

### Population structure

**Fig 2:**
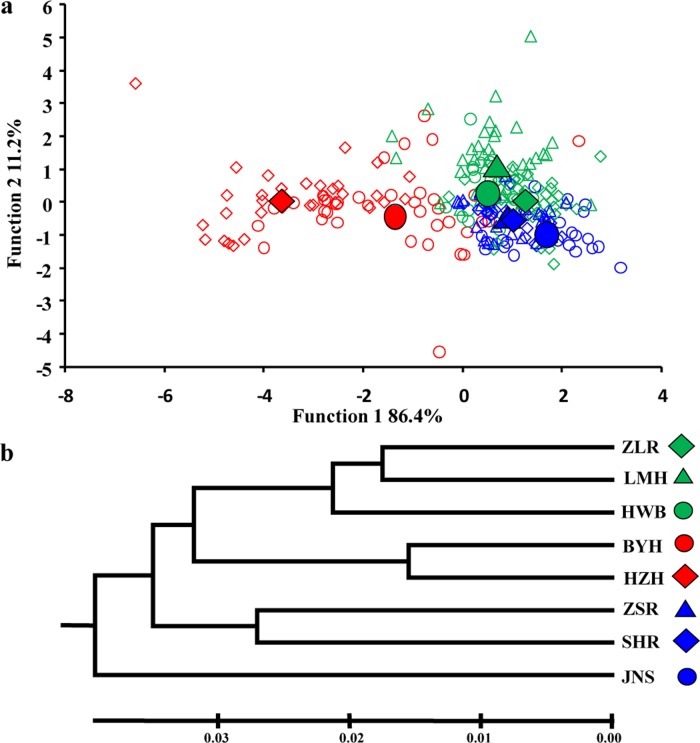
Relatedness among eight *D*. *galeata* populations from Eastern China (based on up to 15 microsatellite loci). (a) Discriminant analysis on FCA scores (four factorial axes) was used to discriminate among groups of individuals from eight lakes. Shown are values from the first two discriminant functions per individual and eight group centroids (full symbols) representing the eight lakes. The predicted lake membership of individuals in open symbols. (b) UPGMA clustering of Nei’s genetic distances.

**Fig 3:**
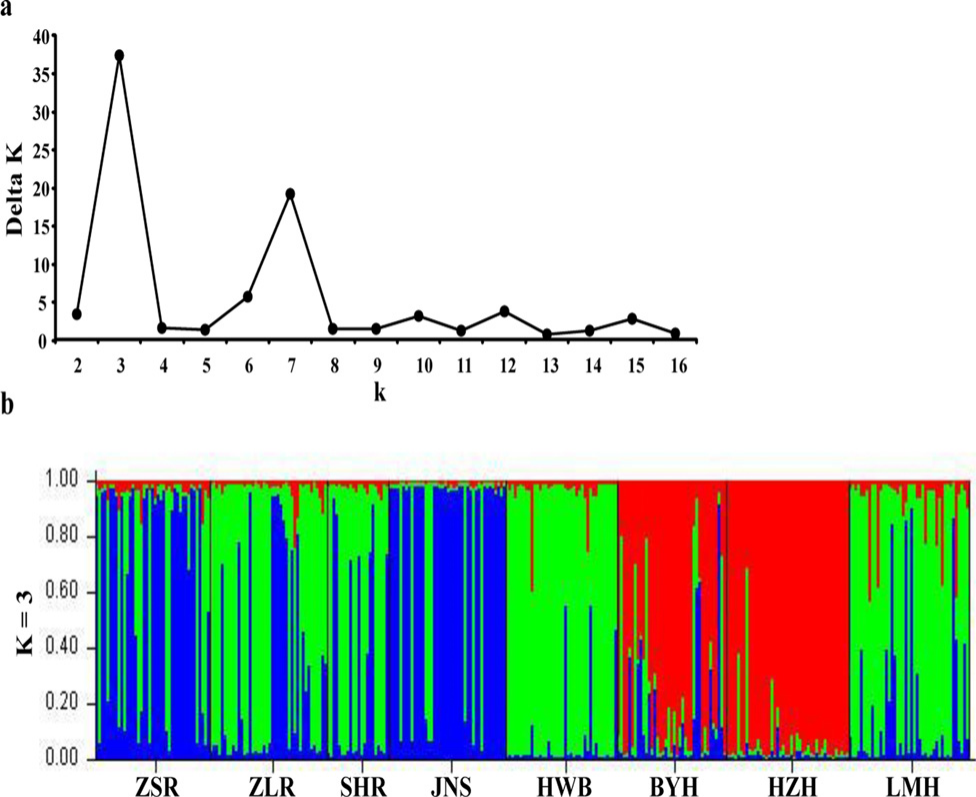
Results from a Bayesian assignment analysis of microsatellite data in STRUCTURE. a) Delta *K* values as a function of *K*, according to the method reported in Evanno et al. (2005). The highest peak was for *K* = 3; b) Assignment of each individual to any of the three groups (i.e. *K* = 3).

In the FCA run on 250 unique MLGs (allowing missing data at up to four loci) originating from the eight Chinese populations, the first two factorial axes explained 10.46% of the variance in the data (Fig. 2a). Discriminant analysis on all extracted factorial axis scores (eight lakes as grouping factor) resulted in four functions. The first function with an eigenvalue of 2.97 already explained 86.4% of the variance, while the second function explained additional 11.2%. Group centroids based on the first two functions revealed that BYH and HZH were separated from the other six populations by scores on function 1 (Fig. 2a). The close neighbourhood of group centroids from the latter six lakes with respect to function 1 revealed that the likelihood of misclassification of individuals by MLG to a wrong population is high. Accordingly, only 48.0% of individuals are correctly reclassified to their lake of origin. The separation of BYH and HZH from other populations was further confirmed by the pattern in the UPGMA tree (Fig. 2b) and the clustering in STRUCTURE (Fig. 3). Additionally, the other six populations were split into two groups, by both methods (Figs. 2b and 3), a slight differentiation was also visible regarding the group centroids of function 2 in DFCA (Fig. 2a). Accordingly, in terms of STRUCTURE, *K* = 3 was the best fit (Fig. 3a). A single cluster, containing BYH and HZH, emerged by all methods, but because the resolution among the remaining six populations was low, STRUCTURE identified one additional cluster of two lakes and another cluster of four lakes while UPGMA and DFCA weakly resolved among two clusters of three lakes each. Interestingly, the second highest peak of *ΔK* (i.e. *K* = 7, Fig. 3a), implies that further substructure can be found among seven units only, instead of among all eight distinct lakes (lakes BYH and HZH could not be separated at this level; data not shown). This result suggests, that lake-specific substructure exists but is only of minor importance, while the major differentiation is among three groups of lakes supported by all analyses. Notably, the results from STRUCTURE, DFCA and UPGMA suggest that besides populations from the two neighbouring lakes HZH and BYH, most populations did not cluster by geographic region (S1 Fig.).

To further explore the lake-specific substructure, we used only individuals with complete MLGs (i.e. 264 individuals). Among those, 201 unique MLGs were detected. Identical MLGs were detected only within, but not across the lakes. Individuals possessing identical MLG could be treated as descendants from the same clonal line, as *P*_sex_ values were lower than 10^-4^, rejecting the hypothesis that individuals sharing identical MLG were of sexual origin. The relative clonal richness ranged from 0.47 to 1.00, and the clonal diversity varied between 0.86 and 0.97 (Table 1). The clonal richness value of 1.00 (detected in three populations) indicates that all the individuals possessed different MLGs. Because in most lakes only few individuals were found to share identical MLGs (only three populations had a higher level of clonality: HWB, JNS and ZSR; Table 1), we provide results from population genetic analyses although the assumption of the tests are violated to some degree. Genetic differentiation was significant for all tested pairs of *D*. *galeata* populations, and *F*_ST_ values ranged from 0.06 to 0.25 (averaged over all loci). In contrast to the findings from STRUCTURE also populations from HZH and BYH were slightly differentiated (*F*_*ST*_ = 0.06, *P* < 0.001) which is consistent with significant differences of respective mean discriminant scores on function1 (t = 6.274, n = 72, *P* < 0.001). A significant deviation from Hardy-Weinberg equilibrium was detected in only two of the eight populations, HZH and JNS, with *F*_*IS*_ of -0.29 and -0.35, respectively (Table 1).

## Discussion

The fact that only one species (*Daphnia galeata*) from the *D*. *longispina* complex was present in the investigated area is surprising. By contrast, a report from the 1970s claimed that several different species of the *D*. *longispina* complex coexisted in China [24]. Specifically, *D*. *galeata* coexisted with *D*. *longispina* in the lakes of Eastern China (e.g. Jiangsu Province) [24]. However, the assignment of species was based only on morphology [24]. *D*. *galeata* and *D*. *longispina* have similar body shapes, leading to errors in species assignment by morphological classification compared to molecular methods [51]. Thus, the *D*. *longispina* phenotypes reported from the 1970s might have been incorrectly identified. However, we may also have failed to detect other species present in low numbers due to under-sampling. In the current study, lakes were sampled for zooplankton only once, whereas seasonal replacement of different *Daphnia* species has been observed in long-term field surveys (e.g. [52–54]). Nonetheless, the fact that some of the lakes in the present study were sampled in spring, whereas others were sampled in autumn, reduces the possibility that other species from the *D*. *longispina* complex which could otherwise be adapted to one specific season were missed. Several studies in Europe and Japan showed, using genetic techniques, that *D*. *galeata* often coexists with other species from the *D*. *longispina* complex, and produces interspecific hybrids (e.g. [8,10]). Therefore, we would also expect to detect hybrids in the studied lakes if other species from the *D*. *longispina* complex also occurred in the lakes, but this was not the case.

The observed exclusive presence of *D*. *galeata* might be driven by the ecological conditions, possible being optimal for this species. Recent measurements of nutrients showed that all eight lakes are eutrophic (Wei *et al*., unpublished data), and *D*. *galeata* is indeed known to be adapted to high trophy levels. Specifically, *D*. *galeata* was able to invade lakes when they became eutrophic (e.g. [30]), and also showed higher performance under eutrophic conditions in a laboratory experiment [55]. Thus, in the studied area, *D*. *galeata* could have completely replaced other resident species from the *D*. *longispina* complex, when the lakes became eutrophic. Although the twelve sampled lakes which did not contain *Daphnia* are also eutrophic (Wei *et al*., unpublished data), all these habitats (except Gaoyou lake) are very small and had experienced recent and dramatic ecological changes, such as a significant reduction in area due to the upstream establishment of the Three Gorges Dam. This could have resulted in the extinction of *D*. *longispina* populations.

Most *D*. *galeata* populations investigated here showed high relative clonal richness (average = 0.78) and clonal diversity (average = 0.94), independent of the time of sampling (spring or autumn). This finding is in agreement with results of a recent study, where the clonal richness of *D*. *galeata* populations remained stable over the course of a year [56]. One reason for the high clonal richness of the studied populations could be that sexual reproduction recreates genotypic variation, and that clonal selection has little effect on the genetic diversity. Indeed, we detected no deviations from Hardy-Weinberg equilibrium in six out of eight *D*. *galeata* populations, suggesting frequent sexual reproduction in this system. This finding is consistent with results from previous studies on the *D*. *longispina* complex, where genotype frequencies were in good agreement with Hardy-Weinberg expectations, as derived from allozyme markers (e.g. [57,58]) and recently confirmed by microsatellites [34].

Based on three different methods (Bayesian assignment, discriminant analysis based on results from factorial correspondence analysis and cluster analysis based on genetic distances) the *D*. *galeata* populations from two neighbouring lakes (i.e. BYH and HZH) were genetically very similar, whereas other lakes were divided into two subgroups. Potentially, this is due to a common history of the two neighbouring lakes in the past. In former times, a connecting river allowed the exchange of MLGs between BYH and HZH in case of flooding. This could explain the similarity among the two populations, although the lakes have been artificially isolated since more than ten years, by a road constructed on a dam. The question why the other six lakes are divided into two subgroups cannot be answered from our study.

This is the first study examining the genetic structure of populations of the *D*. *longispina* complex in Eastern China. We found that *Daphnia* individuals belonging to this complex were present in eight out of twenty lakes sampled. All eight lakes were inhabited exclusively by one species, *D*. *galeata*, in contrast to a historical study reporting more species. Our finding calls for further studies to explore the genetic architecture of *Daphnia* diapause egg banks produced over the last decades in those lakes (such as in [30,31]). This will allow the detection of a potential biodiversity loss in these aquatic systems, caused by anthropogenic impacts, such as eutrophication.

## Supporting Information

S1 Fig Geographical position of the eight *D*. *galeata* populations examined in the present study (see Table 1 for lake abbreviations). The genetic composition of populations relates to the percentage of individuals assigned to three genetic groups, as defined by STRUCTURE analysis (see also Fig. 3).
(TIF)

S1 Table List of the twelve sampling sites in Eastern China, where no individuals from the *Daphnia longispina* complex were detected. All lakes were sampled in late spring of 2013.
(DOCX)
