# Supplementary figures and images for: Genetic Structure of Daphnia galeata Populations in Eastern China
Source: PLoS One. 2015 Mar 13;10(3):e0120168. doi: 10.1371/journal.pone.0120168 (PMC4358959; doi:10.1371/journal.pone.0120168)

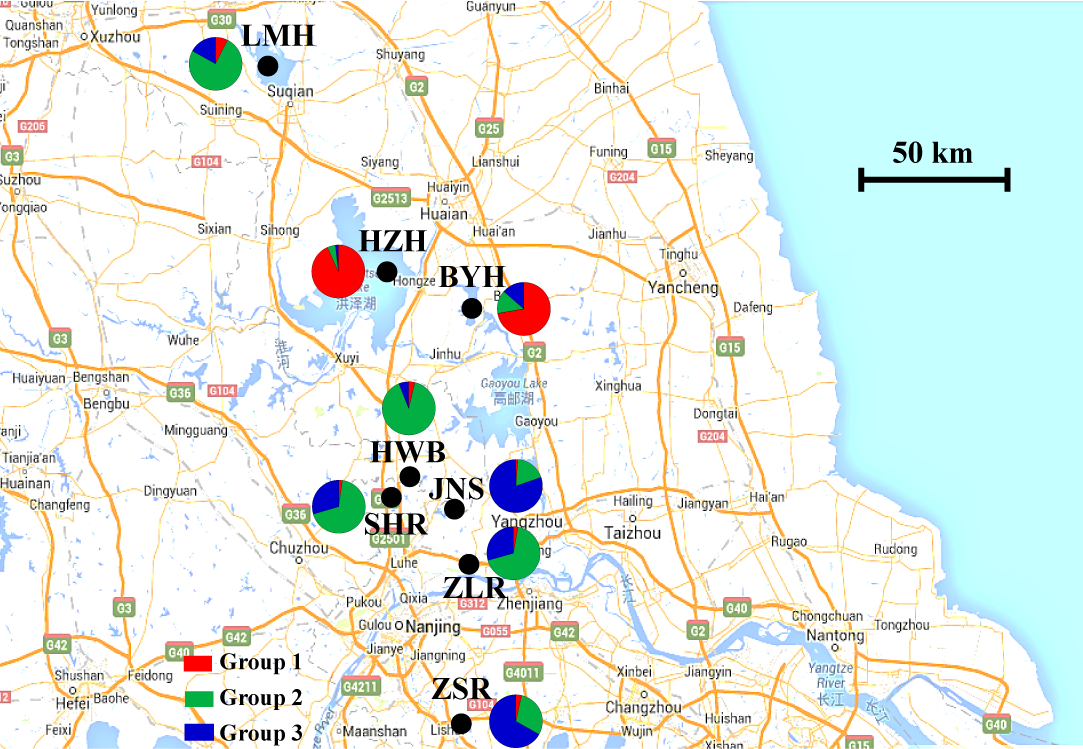

Supplement: S1 Fig — The genetic composition of populations relates to the percentage of individuals assigned to three genetic groups, as defined by STRUCTURE analysis (see also Fig. 3). (TIF) [file pone.0120168.s001.tif]
