# Supplementary material for: Genetic Structure of Daphnia galeata Populations in Eastern China
Source: PLoS One. 2015 Mar 13;10(3):e0120168. doi: 10.1371/journal.pone.0120168 (PMC4358959; doi:10.1371/journal.pone.0120168)
Supplement: S1 Table — All lakes were sampled in late spring of 2013. (DOCX) [file pone.0120168.s002.docx]

S1_Table. List of the twelve sampling sites in Eastern China, where no individuals from the *Daphnia longispina* complex were detected. All lakes were sampled in late spring of 2013.

| Lake | Latitude, longitude | Surface area (km^2^) | Origin |
| --- | --- | --- | --- |
| Dongfanghong | 32°21′, 119°00′ | < 1 | Artificial |
| Daguantang | 32°26′, 118°47′ | < 1 | Artificial |
| Gaoyou | 32°53′, 119°15′ | 674.7 | Natural |
| Fuwan | 32°26′, 118°44′ | < 1 | Artificial |
| Houliuba | 32°26′, 118°47′ | < 1 | Artificial |
| Huangshan | 32°29′, 118°54′ | < 1 | Artificial |
| Shahequ | 32°20′, 119°02′ | < 1 | Artificial |
| Sanyou | 32°21′, 119°00′ | < 1 | Artificial |
| Tanglou | 32°23′, 119°23′ | < 1 | Artificial |
| Tangzhuang | 32°26′, 118°46′ | < 1 | Artificial |
| Yuetang | 32°24′, 119°05′ | 1.4 | Artificial |
| Zhaoqiao | 32°31′, 118°52′ | < 1 | Artificial |
